# One Tree to Link Them All: A Phylogenetic Dataset for the European Tetrapoda

**DOI:** 10.1371/currents.tol.5102670fff8aa5c918e78f5592790e48

**Published:** 2014-08-08

**Authors:** Cristina Roquet, Sébastien Lavergne, Wilfried Thuiller

**Affiliations:** Evolution, Modeling and Analysis of Biodiversity, CNRS LECA, Université Joseph Fourier, Grenoble, France; Evolution, Modeling and Analysis of Biodiversity, CNRS LECA, Université Joseph Fourier, Grenoble, France; Evolution, Modeling and Analysis of Biodiversity, CNRS LECA, Université Joseph Fourier, Grenoble, France

## Abstract

Since the ever-increasing availability of phylogenetic informative data, the last decade has seen an upsurge of ecological studies incorporating information on evolutionary relationships among species. However, detailed species-level phylogenies are still lacking for many large groups and regions, which are necessary for comprehensive large-scale eco-phylogenetic analyses. Here, we provide a dataset of 100 dated phylogenetic trees for all European tetrapods based on a mixture of supermatrix and supertree approaches. Phylogenetic inference was performed separately for each of the main Tetrapoda groups of Europe except mammals (i.e. amphibians, birds, squamates and turtles) by means of maximum likelihood (ML) analyses of supermatrix applying a tree constraint at the family (amphibians and squamates) or order (birds and turtles) levels based on consensus knowledge. For each group, we inferred 100 ML trees to be able to provide a phylogenetic dataset that accounts for phylogenetic uncertainty, and assessed node support with bootstrap analyses. Each tree was dated using penalized-likelihood and fossil calibration. The trees obtained were well-supported by existing knowledge and previous phylogenetic studies. For mammals, we modified the most complete supertree dataset available on the literature to include a recent update of the Carnivora clade. As a final step, we merged the phylogenetic trees of all groups to obtain a set of 100 phylogenetic trees for all European Tetrapoda species for which data was available (91%). We provide this phylogenetic dataset (100 chronograms) for the purpose of comparative analyses, macro-ecological or community ecology studies aiming to incorporate phylogenetic information while accounting for phylogenetic uncertainty.

## Introduction

The use of phylogenetic data into ecological analyses has grown rapidly in the last decades, giving rise to new disciplines such as community phylogenetics which incorporate information on species relatedness into the study of community structure^1^^,^ 2, as well as to studies of large-scale distribution of species and their phylogenetic diversity^3^^,^ 4. Additionally, the integration of ecological and evolutionary information holds promise to improve ecological forecasting in the current context of climate and land change and biodiversity loss^5^^,^ 6. Since the pioneering work of Dan Faith^7^, conservation biology has long recognized the importance of considering phylogenetic diversity as a relevant feature for conservation^8^^,^ 9. The EDGE framework is, in this regard, an important initiative that combines the evolutionary distinctiveness of species (i.e. the evolutionary contribution of a species to the tree of life) with globally endangered risk assessment to derive conservation priorities^10^. Recent works have also focused on how future climate and land use change could further jeopardize the tree of life in certain parts of the world^11^^,^ 12.

To foster the developments of these emergent fields and timely questions, detailed and broadly sampled phylogenetic hypotheses are needed to appropriately integrate evolutionary information into ecological and conservation studies. Recent phylogenomic studies have improved our understanding of the evolutionary relationships within the main Tetrapoda groups, especially at high levels such as families and orders. For instance, Roelants and colleagues^13^ clarified the relationships between global amphibians at the family level, while Pyron et al.^14^ performed a similar achievement on Squamata, sampling all families and sub-families. Concerning birds, Hackett et al.^15^ elucidated the inter-ordinal relationships of extant birds, and a later study^16^ confirmed the partly controversial results found by Hackett and colleagues. Despite these achievements, we still lack detailed species-level phylogenies for such groups. Moreover, there is a lack of phylogenies for particular regions (but see 17), as systematists mainly focus on building species-level phylogenies for entire clades. Although it is of obvious interest, research areas such as community phylogenetics and conservation planning do not specifically require complete taxonomic sampling, but rather complete spatial, or biogeographic, sampling. In order words, ecological studies that wish to integrate evolutionary data usually require a phylogenetic hypothesis for the entire species pool under study, which might be along a specific gradient^18^ or a continental scale assessment^11^^,^ 19 . For instance, incorporating phylogenetic diversity in reserve design or gap analysis only require a complete phylogenetic tree for the entire group with the region of interest (see for example 19, 85). It should however be noted that since the complete coverage only concerns Europe, estimates of phylogenetic uniqueness are therefore biased and should be accounted for in the analysis of the data (e.g. 86).

For that purpose, we here construct and provide a phylogenetic dataset for all Tetrapoda species that occur in the entire European sub-continent (including Turkey) built on relevant phylogenetic data in Genbank and consensus tree knowledge, based on a supermatrix-supertree mixed approach^20^ . We also check the congruence of the phylogenies obtained with previous evolutionary studies.

## Methods

**Squamata and Testudinae**

****The list of European Squamata species was extracted from Maiorano et al^21^. DNA sequences of 7 nuclear (BDNF, c-mos, NT3, PDC, R35, RAG-1, RAG-2) and 6 mitochondrial loci (12S, 16S, COI, cytB, ND2, ND4) were downloaded from Genbank with PHLAWD^22^. These regions have been shown to be useful for phylogenetic inference in previous studies of squamates according to Pyron et al^14^. Only 16 species of a total of 239 had no molecular data available in Genbank. In addition to Squamata species, we included 3 levels of outgroup taxa: *Sphenodon punctata* (closest living relative to Squamata); all 10 species of European turtles, two crocodilians *(Alligator* and *Crocodylus)* and two birds *(Dromaius* and *Gallus);* and finally two mammals *(Mus* and *Pan).* Genbank accession numbers are detailed in Table S1 (Appendix 1).

For each region, DNA sequences were aligned with MAFFT^23^ and checked by eye with Seaview^24^. Ambiguous alignment positions were trimmed with trimAl^25^. All the regions were concatenated in a supermatrix with FASConCAT^26^. The phylogenetic inference analysis was conducted with RaxML v. 7.8.1^27^ using the GTRGAMMA model and employing the rapid hill-climbing algorithm^28^; we searched for 100 Maximum Likelihood trees applying a family tree constraint for squamates based on Pyron et al^14^. Bootstrapping was conducted with 1000 replicates to assess clade support.

The 100 ML trees were dated with penalized-likelihood as implemented in r8s^29^; we constrained 5 nodes based on fossil information extracted from Mulcahy et al.^30^: we set a minimum and a maximum age of 256 and 300 mya respectively for the most recent common ancestor (mrca) of all Reptilia^31^^,^ 32, a minimum and a maximum age of 239 and 250 mya respectively for the mrca of Birds and crocodilians^32^ , a minimum age for the mrca of Lepidosauria of 223 mya^33^^,^ 34, a minimum age of 111 mya for the stem branch of Amphisbaenidae^34^^,^ 35, and a minimum age of 93 mya for the stem branch of Alethinophidia^36^. The best smoothing value was determined by a cross-validation procedure, following 29 .

The data matrix and the phylogenetic tree with the highest likelihood are available in Treebase (accession number: S15708).

**Amphibians**

For Amphibians, we include here the phylogenetic tree constructed for a previous study^19^. The list of European Amphibian species was extracted from Maiorano et al^21^. We retrieved from GenBank sequences of phylogenetic informative regions that were available for at least 30% of the species: 9 mitochondrial (12S, 16S, COI, cytb, ND1, ND2, ND4, tRNA-Leu, tRNA-Val) and 2 nuclear (RAG-1, rho) regions. We found relevant molecular data for all species, but we excluded the two hybrid species* Pelophylax grafi* and *Pelophylax hispanicus*. We included *Xenopeltis unicolor*, *Gallus gallus* and *Mus musculus* as outgroups to root the tree. For each region, alignment was conducted with four programs (Clustal^37^, Kalign^38^, MAFFT^23^ , MUSCLE^39^). The best resulting alignment was selected based on Mumsa^38^, and checked visually. Ambiguous regions of each alignment were removed with trimAl^25^. All regions were concatenated in a supermatrix with FASconCAT. As with Squamata, we obtained 100 ML phylogenetic trees by conducting a phylogenetic inference analysis with RaxML, this time applying a family-level tree constraint based on Roelants et al^13^. A bootstrap analysis was conducted with 1000 replicates to assess clade support.

We dated the 100 ML trees with penalized-likelihood (r8s) using the following fossil data to constrain minimum ages for selected nodes: 155 mya for the crown-origin of salamanders^40^, 170 mya for Bombianura^41^, 250 mya for Batrachia^42^, 110 mya for the split of Pelobatidae and Pelodytidae families^43^, 145 mya for the split of Pelobatidae and Neobatrachia^43^, and 61 mya for the split of Plethodidae and Proteidae^44^. Additionally, we set a minimum and maximum age (312-330 mya) for the split between diaspid (*Gallus gallus*, *Xenopeltis unicolor*) and synapsid amniotes (*Mus musculus*), based on Benton and Donoghue^45^.

The data matrix and the phylogenetic tree with the highest likelihood are available in Treebase (accession number: S13561).

**Birds**

We include here 100 dated phylogenetic trees for 430 species of European breeding birds from Roquet et al^20^. This phylogenetic dataset was built upon sequences retrieved from GenBank for 10 mitochondrial gene regions (12S , ATP6 , ATP8 , COII , COIII , ND1 , ND3 , ND4 , ND5 , ND6) and six nuclear ones (28S , c-mos, c-myc , RAG-1 , RAG-2 , ZENK). The alignment procedure was the same as for Amphibians. We also performed 100 ML phylogenetic inference searches and standard bootstrapping (1000 replicates) with RaxML, applying a tree constraint at the ordinal level based on Hackett et al^15^. The 100 trees were dated with penalized likelihood (r8s) applying fossil calibrations for 14 clades (Table S2, Appendix 2). The best ML tree can be found in Treebase (study number 10770).

**Mammals**

The phylogenetic data here included for mammals is based on the super-tree of Fritz and colleagues^46^; concretely, we extracted the resampled dataset of 100 fully resolved phylogenetic trees from Kuhn et al.^47^, where polytomies of the super tree from Fritz et al.^46^ were randomly resolved applying a birth-death model to simulate branch-lengths. Then, for each tree, we replaced the Carnivora clade with the update performed on a recent study^48^, which provides a better resolution and increases the sampling from 252 sp to all Carnivora species (286 sp). Later, we removed all non-European species. These modifications of the phylogenetic trees were done with the R package *ape.*

**Phylogenetic inference**

As stated before, for each taxon group except mammals we have conducted 100 ML inferences with RAxML. Every inference begins with a different starting tree, which is built by adding sequences one by one in random order, identifying their optimal location on the tree under the parsimony optimality criterion. Since sequences are added in random order, it is very likely that a different starting tree is generated at every search^87^ ,88. RAxML searches were then performed with the method "lazy subtree rearrangement" (a variant of subtree prunning and regrafting method) under a ML framework. Like all heuristic search strategies, the Maximum Likelihood search strategy employed by RAxML is not guaranteed to find the most probable tree of the tree-space, and because of that, it is important to conduct multiple searches from different starting trees. To check if all the searches converged on trees with similar likelihoods, we performed the Shimodaira-Hasegawa test^89^ (SH) implemented in RAxML. In all cases, the likelihoods of the trees of a same group of taxa were not significantly different (p < 0.01). This increases our confidence on the trees found being close/similar to the most likely tree, and that the trees obtained do not result from the algorithm getting stuck in a local optima.

**Supertree construction**

The trees cited above were combined, after pruning the outgroups, by joining them with the R package *ape*; to do so we set divergence ages between these main groups based on the information retrieved in the webpage Timetree 49: the divergence age between mammals and sauropsids (i.e. birds, turtles and squamates) was set to 324 mya, and the divergence age between amphibians and the rest of the groups was set to 361 mya. To build the final tetrapod tree, we randomly selected one tree from each of 100 trees available for each group. We repeated this approach 100 times to get 100 realisations of the tetrapod tree. These combinations were done randomly since the likelihoods of the trees of each group were not significantly different according to the SH test. The 100 dated trees of each group and the 100 dated supertrees for all European Tetrapoda are available from the Dryad digital repository (DOI: X).

## Results and Discussion

**Squamata**

The study of Pyron et al.^14^ constituted a major advance in our understanding of the phylogenetic relationships between the main lineages of Squamata. Their study had a broad taxonomic and molecular sampling: they included members of all currently recognized families and subfamilies, for which 7 nuclear and 5 mitochondrial loci were analysed. Here, we took profit of the knowledge derived from that study by incorporating a tree constraint to the family level based on their results. We also performed the analysis without the tree constraint (results not shown); the results were congruent with the first analysis, but the lack of a family-tree constraint yield low bootstrap (BS) support for the deepest nodes.

Our phylogenetic results are largely congruent with those of Pyron and colleagues^14^. We have similar levels of strong nodal supports except for the relationships between genera of Lacertidae; 67.8% of the nodes had a strong support (BS > 70%, Fig. 1, Appendix 3) and 13.1% of the nodes had a moderate support (BS 50-70%). In accordance with their study, we detected that some genera are not monophyletic: *Ablepharus* (Scincidae), *Cyrtopodion* (Gekkonidae), *Zamenis* (Colubridae). We also found strong evidence that *Hierophis* and *Dolichophis* (Colubridae) are not monophyletic genera, as* D. cypriensis* (which was not included in 14) is nested within *Hierophis* with a 100% BS.

Available dating studies on Squamata differ considerably on age estimates. For instance, a recent study^30^ estimated the squamate crown group to be c. 180 mya, while two other studies estimated the same group to be c. 240 mya^50^^,^ 51. Our estimates of divergence times are in general roughly similar to those of Kumazawa's study^50^. It has been suggested^30^ that the use of only mitochondrial regions (which is not the case here) may bias the results towards older ages, but anyway differences in methodology and in taxon and molecular sampling make difficult to identify all the causes of those discrepancies.

**Amphibians**

The phylogenetic inference analysis for the amphibians yielded a particularly robust topology: 83.5 % of the nodes showed a strong support (BS>70%, Fig. 2, Appendix 3). Supported nodes of our ML trees were congruent with previous phylogenetic studies^52^^,^ 53 ^,^ 54 ^,^ 55 ^,^ 56 ^,^ 57. Concerning the divergence age estimates, we obtained younger ages for the deepest nodes compared to the work of Roelants and colleagues^13^, for instance, Batrachia was estimated with r8s to be c. 330 mya in that study, while we estimated it to be c. 300 mya; in contrast, we retrieved older ages for the shallowest nodes (e.g. we estimated that the divergence between *Salamandra* and *Pleurodeles* occurred 100 mya, Roelants and colleagues estimated it to have occurred c. 75 mya). These differences might be linked to the difference in molecular and taxon sampling: Roelants et al. sampled only one species per genera; and several families that were included in their work are not present in Europe and thus were not included in our supermatrix.

**Birds**

Supported nodes of our ML trees are congruent with previous phylogenetic studies (Anseriformes: Donne-Goussé et al. 58, Eo et al.^59^, Gonzalez et al.^60^; Galliformes: Gutierrez et al.^61^ , Dimcheff et al.^62^, Crowe et al.^63^, Kimball et al.^64^, Kriegs et al.^65^, Lislevand et al.^66^; Gruiformes: Fain et al.^67^; Procellariiformes: Penhallurick and Wink^68^; Ardeidae: Sheldon et al.^69^ ; Accipitridae: Lerner and Mindell^70^, Griffiths et al.^71^ ; Charadriiformes: Paton et al.^72^ , Thomas et al.^73^, Pons et al.^74^ , Bridge et al.^75^ , Paton and Baker^76^ , Fain and Houde^77^; Passeriformes: Alström et al.^78^, Nguembock et al.^79^, Treplin et al.^80^; Piciformes-Coraciiformes: Johansson et al.^81^, Benz et al.^82^; Strigiformes: Wink et al.^83^); 68.7% of the nodes had a strong BS support (BS>70%, Fig. 3, Appendix 3), and an additional 12.4% had a moderate support (BS=50-70%). Divergence age estimates were, in general, congruent with those obtained by Brown et al^84^.

**Mammals**

The modification of the most recent mammals supertrees available on the literature^47^ with the update of Carnivora clade^48^ allowed to increase the phylogenetic resolution (only nine polytomies remain in the updated Carnivora clade) and to have a higher species sampling.

**The importance of accounting for phylogenetic uncertainty**

Phylogenetic information is sometimes incorporated in ecological analyses based on a single phylogenetic tree, assuming the tree is known without error. Any phylogenetic tree estimate will probably not be an exact representation of the true phylogeny due to possible bias or uncertainties such as molecular and taxon sampling, sequence alignment, homoplasy, or long-branch attraction^90^ ^91^. For all these reasons, it is important to include phylogenetic uncertainty in order to avoid overestimating our confidence in subsequent analyses (i.e. obtaining too narrow confidence intervals). This type of uncertainty can be accounted for in two ways: with a single consensus tree (in which unsupported nodes are collapsed into polytomies), or running the analyses with a range of trees and later summarising the results^11^, ^93^. The first approach (i.e. consensus tree) may not be preferable, as polytomies can influence the results of tree-based statistical analyses (e.g. see 92 for the influence of phylogenetic resolution on several community phylogenetics indices), and do not allow to fully explore the variation in ecological patterns resulting from phylogenetic uncertainty. Moreover, not all methods have been adapted to allow for polytomies, some of them require completely bifurcating trees (e.g. the EDGE index^10^). For all these reasons, we highly recommend to account for phylogenetic uncertainty by including a set of high-probability trees.

**Data availability statement**

The 100 dated supertrees for all European Tetrapoda and the 100 dated trees of each taxon group (amphibians, birds, mammals, squamates and turtles) are available from the Dryad digital repository (DOI: X).

## Conclusion

We provide here a phylogenetic dataset constituted of 100 chronograms of European Tetrapoda species as a tool for ecological studies that aim to incorporate an evolutionary perspective, and for phylogenetic conservation assessment. This phylogenetic dataset is in general agreement with previous studies, and we expect it to be coarsely approximate with the "true" Tetrapoda evolutionary tree. Instead of providing the best ML tree for every group, we provide 100 trees (available on Dryad repository), as computing analyses with several trees allows taking in account phylogenetic uncertainty. Regarding the taxonomic sampling, the big majority of species are included (91%). On the other side, some molecular regions have low sampling, thus, this dataset will be useful until substantial amount of molecular data becomes available for a considerable number of species.

## Competing Interests

The authors have declared that no competing interests exist.

## Figures and Tables

**Phylogenetic tree of European squamates and turtles:**
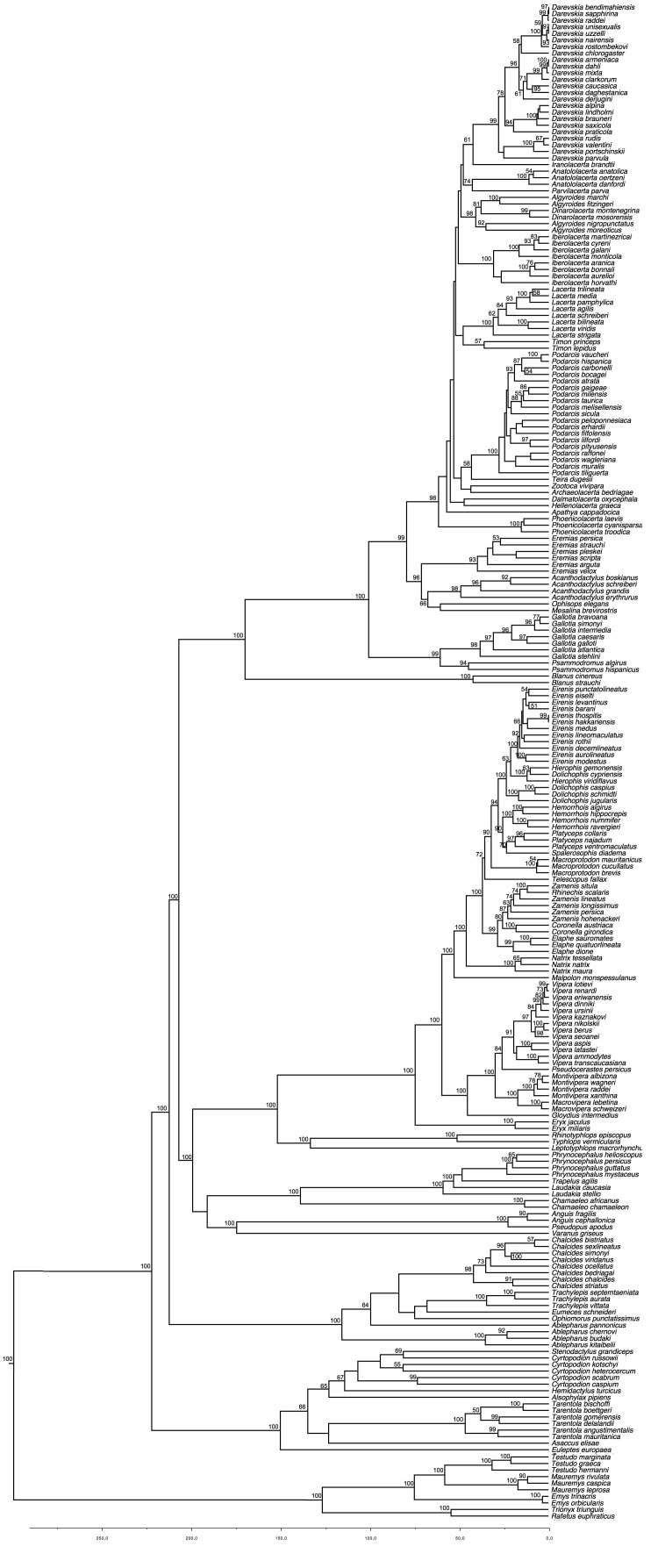
Phylogenetic tree obtained by maximum likelihood inference with RaxML (outgroups have been removed). Numbers above branches indicate bootstrap values.

**Phylogenetic tree of European amphibians:**
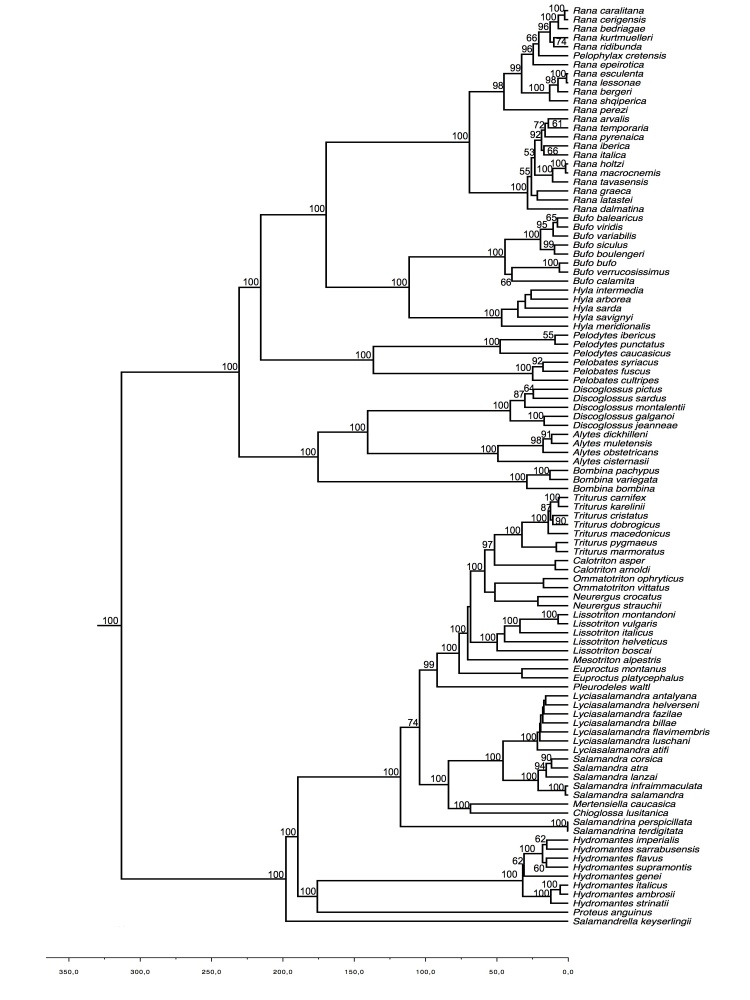
Phylogenetic tree obtained by maximum likelihood inference with RaxML (outgroups have been removed). Numbers above branches indicate bootstrap values.

**Phylogenetic tree of European birds:**
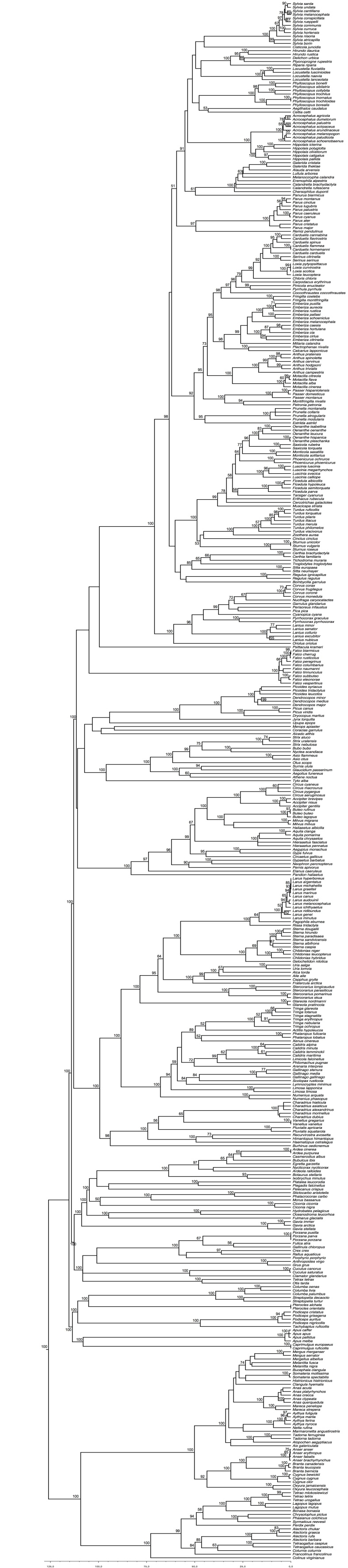
Phylogenetic tree obtained by maximum likelihood inference withRaxML. Numbers above branches indicate bootstrap values.

## Appendix 1

### Genbank accession numbers for squamates and turtles

Download PDF

## Appendix 2

### Calibration of the bird phylogenetic trees

**Table:** **Table S2**. Summary of the fossil data used for calibration of the phylogenetic trees of birds. All fossils were used as minimum age constraints, except *, which was used as a maximum age.

Clade	Age	Stem/Crown	References
Anseriformes	66	Stem	Clarke et al. (2005)
Ingroup	125*	Stem	Zhou (2004)
Apodiformes	53	Stem	Mayr (2003)
Upupidae+ Picidae	47.05.00	Stem	Mayr (2000)
Coraciidae+ Meropidae	47.05.00	Stem	Mayr & Mourer-Chauviré (2000)
Pandionidae	37	Crown	Harrison & Walker (1976)
Sulidae	33	Stem	Mayr (2002)
Pteroclidae	30	Stem	Mourer-Chauviré (1993)
Rallidae	33	Stem	Mayr & Smith (2001)
Scolopaciade	33	Stem	Olson (1985)
Strigidae	58	Stem	Vickers-Rich & Bohaska (1976)
Procellariidae	23	Stem	Olson (1985)
Sulidae	33	Stem	Mayr (2002)
Burhinidae	18	Stem	Bickart (1982)

**Additional references**

Clarke JA, Tambussi CP, Noriega JI, Erickson GM, Ketcham RA. 2005. Definitive fossil evidence for the extant avian radiation in the Cretaceous. Nature 433: 305-308.

Harrison CJO, Walker CA. 1976. Birds of the British Upper Eocene. Zoological Journal of the Linnean Society 59: 323-351.

Mayr G. 2000. Tiny hoopoe-like birds from the Middle Eocene of Messel (Germany). Auk 117: 968-974.

Mayr G. 2002. A skull of a new pelecaniform bird from the Middle Eocene of Messel, Germany. Acta Palaeontologica Polonica 47: 507-512.

Mayr G. 2003. Phylogeny of early Tertiary swifts and hummingbirds (Aves: Apodiformes). Auk 120: 145-151.

Mayr G, Mourer-Chauviré C. 2000. Rollers (Aves: Coraciiformes s.s.) from the Middle Eocene of Messel (Germany) and the Upper Eocene of the Quercy (France). Journal of Vertebrate Paleontology 20: 533-546.

Mayr G, Smith R. 2001. Duck, rails, and limicoline waders from the lowermost Oligocene of Belgium. Geobios 34: 547–561.

Mourer-Chauviré C. 1993. Les gangas (Aves, Columbiformes, Pteroclidae) du Paléogène et du Miocène inférieur de France. Palaeovertebrata 22: 73-98.

Olson SL. 1985. The fossil record of birds. In: Farner D (ed.), Avian Biology. Academic Press, pp. 80–238.

Vickers-Rich P, Bohaska DJ. 1976. The world’s oldest owl: A new 1944 strigiform from the Paleocene of Southwestern Colorado. 1945 Smithsonian Contributions to Paleobiology 27:87–93.

Zhou Z. 2004. The origin and early evolution of birds: discoveries, disputes, and perspectives from fossil evidence. Naturwissenschaften 91: 455-471.
